# Surgical Management of Retroperitoneal Liposarcoma: Opportunities for Multimodality Treatment, Including Systemic Therapy

**DOI:** 10.1002/cam4.71129

**Published:** 2025-08-08

**Authors:** Steven Sun, Kenneth Cardona, William W. Tseng

**Affiliations:** ^1^ Division of Surgical Oncology, Department of Surgery City of Hope National Medical Center Duarte California USA; ^2^ Division of Surgical Oncology, Department of Surgery Winship Cancer Institute, Emory University School of Medicine Atlanta Georgia USA

**Keywords:** dedifferentiated, liposarcoma, multimodality surgery, retroperitoneal sarcoma, surgery, well‐differentiated

## Abstract

**Introduction:**

Soft tissue sarcomas are a diverse group of rare cancers, with approximately 15%–20% found in the retroperitoneum. Liposarcomas (LPS) make up approximately half of all retroperitoneal (RP) sarcomas, with most cases classified as either well‐differentiated (WD) or dedifferentiated (DD). DD LPS is more aggressive, with a higher local recurrence rate and risk of distant metastasis compared to WD LPS. The purpose of this review is to outline surgical management of RP LPS and highlight the multimodal treatment strategies for both primary and recurrent disease, along with considerations for their effective implementation.

**Methods:**

The current medical literature was reviewed for studies focused on retroperitoneal liposarcoma and its treatment with surgery, radiation, and chemotherapy. The data was interpreted and compiled in the context of expert clinical experience.

**Results:**

Along with histopathologic analysis, tumor biology can inform patient prognosis. Surgery, the standard treatment for RP LPS, can be either curative or palliative. In primary disease, an attempt should be made to achieve wide surgical margins when feasible. Surgery for recurrent disease requires careful timing and an understanding of the potential benefit versus risk. Neoadjuvant radiation therapy can improve local control of RP LPS; however, data supporting the use of neoadjuvant chemotherapy are currently lacking.

**Conclusion:**

Multimodality treatment of RP LPS is complex and requires consideration of tumor biology and extent of disease, along with individual patient characteristics. Multidisciplinary team collaboration is critical for improving outcomes in patients with RP LPS.

## 1 Overview of Retroperitoneal Liposarcoma

Soft tissue sarcomas (STS) are a diverse group of rare cancers arising from the mesenchyme, and they account for < 1% of all malignancies in adults [1, 2]. Approximately 10%–15% of STSs occur in the retroperitoneum [3, 4]. Approximately half of all retroperitoneal (RP) sarcomas are liposarcomas (LPS); these are rare malignant tumors of adipocytic differentiation, which include the well‐differentiated (WD), dedifferentiated (DD), myxoid, or pleomorphic subtypes [1, 5]. WD and DD make up the majority of LPS cases and, along with leiomyosarcoma, are the most frequently observed RP sarcoma types [4, 5, 6].

WD tumors recapitulate the histologic characteristics of mature fat and are typically slow‐growing masses with no metastatic potential [4, 5, 7]. In contrast, the more aggressive DD tumors are composed of moderate‐ to high‐grade, non‐lipogenic, undifferentiated cells. Typically, a DD tumor will manifest as a focal non‐fatty component within a WD tumor. Tumors can also be entirely DD without a WD component, although this is rare [4, 5].

In the retroperitoneum, RP LPS tumors can grow to substantial sizes after potentially years of growth. RP LPS tumors can exert a mass effect on surrounding organs or structures (e.g., nerves or blood vessels) and cause symptoms (e.g., pain or venous obstruction). Apart from obvious invasion into organs and structures, the most common symptom in RP LPS is non‐specific abdominal discomfort or distention [3, 4, 8, 9]. Some patients may be asymptomatic; in these cases, their tumors may have been detected by imaging done for another purpose.

Surgery is the standard treatment approach for RP LPS and is the only option that offers a potential cure [10].^1^ Multiple factors should be considered in the surgical management of this disease. There are limited data to date regarding the best approach to utilizing radiation therapy (RT) and chemotherapy in the treatment timeline. This review examines current multimodality treatment strategies—including neoadjuvant systemic therapies—providing valuable insights that may better inform RP LPS management.

## 2 Diagnosis: Imaging and Biopsy

On cross‐sectional imaging, such as computed tomography (CT) or magnetic resonance imaging (MRI), a large, unilateral lipomatous mass of the retroperitoneum should raise suspicion of LPS [11]. WD LPS typically contains > 75% adipose tissue with septations that are thicker than those of normal subcutaneous fat. Unlike WD LPS, DD LPS is characterized by the presence of a non‐lipomatous solid component, within but clearly separate from the remainder of the lipomatous tumor. If DD LPS is suspected, the threshold for obtaining a needle biopsy should be low [12]. However, for WD LPS, clinical guidelines advise that a needle biopsy can be omitted in a subset of patients with suspected RP sarcoma if the imaging is judged pathognomonic by an expert radiologist and no pre‐operative treatment is planned (See Endnote 1) [12]. If a biopsy is performed, an image‐guided core needle biopsy is preferred per clinical guidelines, and open (or minimally invasive) surgical biopsy should be avoided (See Endnote 1).

Definitive diagnosis of RP LPS requires histopathologic examination of the tissue. Tumor heterogeneity poses a challenge during the biopsy process because of the risk of misdiagnosing or undergrading the tumor if tissue is collected from a WD area of a tumor that also has a DD component [13]. For improved diagnostic accuracy, pathologic review should be performed by a pathologist with expertise in STS (See Endnote 1). For DD LPS, the description of histopathologic grade is an important indicator of prognosis [14, 15]. DD LPS can be classified as either grade 2 or 3 according to the French classification system from the Fédération Nationale des Centres de Lutte Contre le Cancer (FNCLCC) [14, 16, 17]. Multicenter data in RP sarcoma suggest that grade alone can stratify patients with DD LPS based on propensity for distant metastasis: Patients with FNCLCC grade 3 had a ~30% incidence at 5 years compared with ~10% for those with grade 2 [15].

In addition to histopathologic assessment, molecular testing may be helpful (See Endnote 1) [13]. Both WD and DD LPS exhibit amplification of chromosome region 12q13‐15, with consistent amplification and overexpression of the murine double minute homolog 2 (*MDM2*) gene, which is considered the main driver gene [7]. There are a number of other genes within the 12q13‐15 region, with amplifications more frequently seen in DD LPS compared with WD LPS [18]. DD LPS also harbors additional mutations, such as 6q23 and 1p32 amplifications, that can be detected; however, these genetic changes are not routinely assessed clinically [18, 19]. Identification of MDM2 amplification helps solidify the diagnosis of LPS; however, the distinction between WD and DD is still largely based on histologic morphology and, frequently, clinical correlation with radiologic findings [19].

Although not universally accepted, positron emission tomography (PET)/CT imaging can guide biopsies and may even be used to distinguish DD areas (See Endnote 1). The diagnostic utility of PET has been explored recently in studies demonstrating high sensitivity and selectivity for identifying histologic subtypes of RP LPS and predicting the aggressiveness of these tumors [20, 21, 22]. Beyond PET, radiomics and CT‐based classifications are emerging tools that may improve diagnosis and assessment of treatment response [17].

## 3 Patient Factors: Baseline Characteristics, Underlying Conditions, Intent of Surgery, and Shared Decision‐Making

The appropriate treatment plan for RP LPS must be determined by considering—as summarized in Table 1 and discussed in detail below—each patient's individual circumstances, in addition to diagnostic data. The necessary and anticipated potential extent of surgery should be carefully weighed against the patient's ability to tolerate surgery, considering any factors that may increase the risk of post‐operative morbidity and mortality [12]. Factors such as advanced age, poor performance status, baseline organ dysfunction (e.g., chronic renal insufficiency), and protein malnutrition should be assessed as part of the development of individual management plans [10, 12, 23]. As an example, malnutrition is often present at diagnosis and may escape clinical detection. Patients with malnutrition have been shown to experience more post‐operative complications and longer hospitalizations [12, 24].

**TABLE 1:** Important considerations for RP LPS treatment planning.

Category	Considerations
Patient characteristics	Age, performance status, baseline organ dysfunction, protein malnutrition
Patient–provider communication	Shared decision‐making discussions when planning treatment
Intent of surgery	Goal of surgery: curative versus palliative
Surgical considerations	Technical aspects of the operation, including resectability
Tumor biology	Histologic subtype (WD vs. DD), primary vs. recurrent disease, multifocality
Multimodality care	Benefits of radiation, systemic therapy, or other modalities

### 3.1 Intent of Surgery

For all patients with RP LPS, it is critical to clarify the intent of surgery—curative versus palliative—up front. This determination should be made through shared decision‐making with the patient during surgical planning. While curative intent should be the goal, in reality, a cure for RP LPS can be quite elusive, given the high risk of local recurrence for both WD and DD disease [25]. One study analyzing data from 22 sarcoma referral centers observed a 6‐year crude‐cumulative incidence of local recurrence from 60.2% for primary WD LPS to 70.9% for FNCLCC grade 3 DD LPS [25]. Generally, patients who experience recurrent disease have worse overall outcomes than those with primary disease, and the risk of recurrence, even after grossly complete resection, does not plateau. Late recurrences up to 15–20 years after resection have been reported [12, 26, 27, 28]. Tools that can be used to predict prognosis after resection may be used to inform treatment planning. One such tool is the nomogram‐based Sarculator app, which was recently updated to incorporate improvements in treatment and outcomes over the past 15 years [29].

In patients for whom surgery is unlikely to be curative, palliative surgery may be performed to address tumor‐related symptoms. LPS may be removed incompletely, partially, or not at all—for example, an internal bypass or colostomy may be done in case of obstruction (See Endnote 1) [30]. Incomplete surgical resection may reduce symptoms and was shown in one study to prolong survival; however, the improvement in outcomes may have been biased by patient selection [30]. The duration and extent of surgical recovery must be carefully considered and balanced with disease biology, given that a more extensive surgery may require an extended post‐operative recovery period during which a patient cannot receive systemic therapy that may have previously offered disease control.

As a critical part of surgical planning, patients should be appropriately informed and have a good understanding about the extent of surgery, recovery, and subsequent impact on quality of life (QoL). In certain cases, surgery may involve multiple teams; it may be helpful for the patient to see consultants from these teams (e.g., vascular surgery) pre‐operatively to better understand specific aspects of the operation [10]. Patients should also have a realistic expectation of both the surgical and oncologic outcomes. Due to the complexity of the decision‐making involved, the patient‐surgeon relationship should be mutually agreeable. Close involvement of the patient's support system and additional follow‐up visits to answer questions prior to surgery are also advisable.

## 4 Key Considerations for Surgical Resection of RP LPS

### 4.1 Technical Resectability

Surgery is the standard treatment for RP LPS, including both the WD and DD subtypes (See Endnote 1). Select outcomes from landmark studies including patients with RP LPS who had surgical resection for their disease (with or without RT or chemotherapy) are summarized in Table 2. To plan surgery, high‐quality cross‐sectional imaging of the abdominopelvic region, specifically contrast‐enhanced imaging (i.e., CT or MRI with contrast, the former being the preferred method), is required (See Endnote 1) [36]. Based on a review of the imaging results, there can be several technical reasons as to why a tumor is deemed unresectable. The primary reason is clear involvement of the celiac or mesenteric vessels [36]. Additional reasons may include diffuse peritoneal disease (sarcomatosis), extensive spine invasion, extensive involvement of the liver hilum, or bilateral kidney involvement.

**TABLE 2:** Key studies reporting RP LPS treatment outcomes.

Article	Study type	Single‐ vs multi‐institution	RP LPS^a^	
			*N*	Key findings
Gronchi et al. [31]	Retrospective	Single	167	Compared with other types of RP sarcoma, patients with LPS showed the greatest benefit with more aggressive surgical approaches in terms of local recurrence.
Bonvalot et al. [32]	Retrospective	Multi: National data	190	*5‐year OS* WD LPS: 0.89 non‐WD LPS: 0.44 *3‐year recurrence rate* WD LPS: 0.34 non‐WD LPS: 0.61 Among patients with RP sarcoma who underwent surgical resection, having WD LPS was associated with a lower recurrence rate and better OS.
Gronchi et al. [15]	Retrospective	Multi: 8 institutions	633	*8‐year OS* WD LPS: > 80%; very few distant metastases DD LPS: 44% Among patients with WD LPS who underwent surgical resection, institutional treatment practices influenced local recurrence rate but not OS
Tan et al. [33]	Retrospective	Single	399	*10‐year disease‐specific death rate* Low‐grade LPS: 25% High‐grade LPS: 53% *5‐year local recurrence rate* Low‐grade LPS: 39% High‐grade LPS: 58% *10‐year distant recurrence rate* Low‐grade LPS: 8% High‐grade LPS: 28% High‐grade LPS (DD, round cell or pleomorphic) was assocated with worse outcomes compared with low‐grade LPS (WD or myxoid) in patients who underwent surgical resection
Haas et al. [34]	Retrospective	Multi: 8 institutions	607	*8‐year local recurrence rate* WD LPS ○ Surgery only: 39.2% ○ Surgery + RT: 11.8% DD LPS (grade 1/2) ○ Surgery only: 56.7% ○ Surgery + RT: 29.0% DD LPS (grade 3) ○ Surgery only: 43.7% ○ Surgery + RT: 29.8% Among patients with LPS who underwent surgical resection, there was no significant benefit for perioperative RT after adjusting for prognostic variables
Raut et al. [25]	Retrospective	Multi: 22 institutions	435	*6‐year OS* WD LPS: 77.1% DD LPS (grade 1/2): 54.6% DD LPS (grade 3): 32.2% Among patients with LPS without distant metastases who underwent surgical resection, second recurrences for LPS were mostly local
Bonvalot et al. [35]	Prospective	Multi: 31 institutions	198	*3‐year abdominal recurrence‐free survival for subjects with RP LPS* (*post hoc analysis*) Surgery only: 65.2% Surgery + RT: 75.7% 10% benefit for addition of RT

Abbreviations: DD, dedifferentiated; LPS, liposarcoma; OS, overall survival; RP, retroperitoneal; RT, radiation therapy; WD, well‐differentiated.

^a^ For studies including multiple tumor types, only data for patients with RP LPS are included here.

The resectability of RP LPS tumors is, however, subjective. Thresholds for technical unresectability may vary depending on the experience and availability of surgical and post‐operative support staff [36]. Overall, the complexity and nuances of the surgical and medical management of this disease merit evaluation of patients in high‐volume sarcoma centers with specific multidisciplinary experience in RP sarcoma [10, 12]. Patients who are deemed unresectable or even borderline resectable may be suitable for non‐surgical therapies up front.

### 4.2 Tumor Biology: WD vs. DD RP LPS

The distinction between WD and DD LPS, which is made by a combination of imaging and histopathologic assessment, is important as this determines clinical behavior and treatment approaches [11]. The presence of DD disease confers a higher risk of both local recurrence and distant metastasis, and the magnitude of this risk must be assessed and considered in the planning and sequencing of treatment [5, 11, 33]. This also extends specifically to surgical planning [11].

Distinguishing between WD and DD LPS may help anticipate the outcome of resection, with the overarching goal of maximizing disease clearance. The histologic subtype may inform whether *en bloc* resection with adjacent organs or structures (e.g., vessels) is required or if organ‐sparing tumor dissection is feasible. WD LPS is considered more likely to have a well‐defined border that is confined to a specific area. The more defined tissue planes may better facilitate dissection of the tumor, especially in primary disease. By contrast, DD LPS is more likely to infiltrate adjacent tissues, making dissection more challenging and frequently necessitating concomitant organ resection.

Histopathologic examination of resected LPS, however, demonstrated adjacent organ invasion in up to 40% of WD tumors [9, 37]. As such, to maximize disease clearance, removal of all RP fat ipsilateral to the tumor from the diaphragm to iliac vessels on the side of tumor origin can be considered, regardless of histologic subtype. Similarly, some sarcoma surgeons would advocate that, again regardless of subtype, *en bloc* organ resection (i.e., extended or compartmental resection) should be performed routinely as long as the morbidity is acceptable (e.g., right colectomy and nephrectomy for right‐sided RP sarcoma).

Importantly, the presence of WD versus DD RP LPS impacts considerations for multimodality treatment, notably therapies to be given in the neoadjuvant setting, before surgery. For resectable tumors that are entirely WD, neoadjuvant RT can be considered. For resectable tumors with a DD component, RT is not recommended, and chemotherapy or clinical trial enrollment, if available, can be considered, particularly for those with aggressive features (e.g., FNCLCC grade 3 or rapid growth suggesting poor disease biology). Recurrence‐free survival after preoperative RT was investigated in the Surgery With or Without Radiation Therapy in Untreated Nonmetastatic Retroperitoneal Sarcoma (STRASS) trial and retrospective STREXIT study, which are discussed below.

### 4.3 Primary vs. Recurrent Disease

The choice of surgical approach and the decision to add non‐surgical therapies are heavily impacted by whether the disease is primary versus recurrent.

**FIGURE 1:**
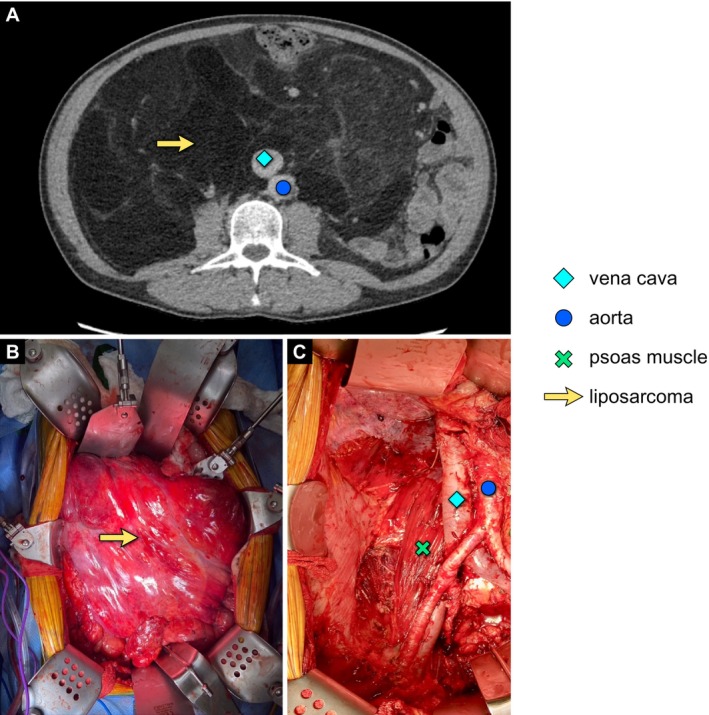
Case study: Primary LPS presentation and treatment. A 65‐year‐old male presented with a right‐sided retroperitoneal well‐differentiated LPS (A). He underwent compartmental resection of tumor with *en bloc* resection of right colon, right kidney, right adrenal gland, and ipsilateral retroperitoneal adipose tissue (B, C). LPS, liposarcoma. Figure courtesy of Kenneth Cardona; MD, FACS.

For primary disease, surgery remains the gold standard and the only possibility to achieve a cure (see case study depicted in Figure 1) [12]. As such, every effort should be made to offer surgery within the limits of resectability and safety. Complete resection with an attempt to achieve wide margins when feasible is the cornerstone of surgical management in primary disease. Piecemeal or partial tumor resection should be strongly avoided because of the risk of tumor rupture, which is associated with early metastasis and worse survival [38].

In a patient with primary RP LPS, one may also consider an extended or compartmental resection [31, 32]. With the extended or compartmental approach—even without obvious tumor involvement—adjacent organs, structures, and surfaces should be resected *en bloc* with the tumor to obtain circumferential negative “soft tissue margins.” [10] Retrospective data demonstrated an overall decreased local recurrence rate compared with standard resection and an improvement in overall survival for low‐ to intermediate‐grade tumors [9, 10, 31, 32]. Whether extended or standard resection is performed, surgery for RP LPS must carefully consider the morbidity potential of organ resection, which is noted to be significantly increased when more than three organs are resected [9, 39]. On an individual basis, preservation of specific organs (e.g., duodenum and head of pancreas) may be attempted to minimize post‐operative morbidity [12].

**FIGURE 2:**
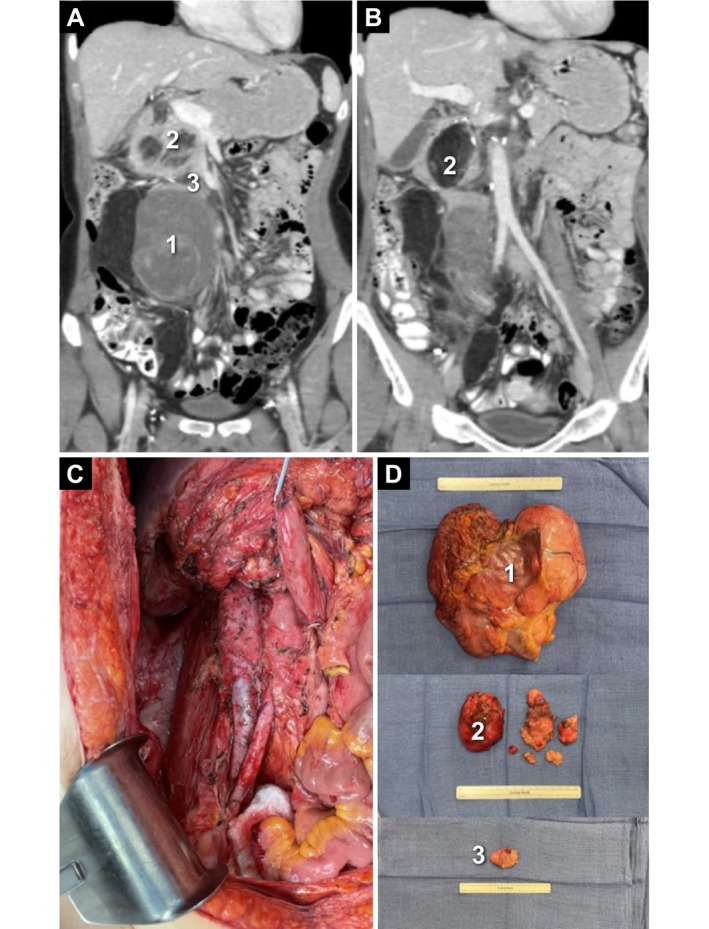
Case study: Recurrent LPS presentation and treatment. A 33‐year‐old female had surgery for primary WD LPS at age 26. She was found to have local recurrence 1.5 years later and subsequently underwent resection with *en bloc* radical nephrectomy, partial colectomy, and total ipsilateral retroperitoneal lipectomy. She developed local recurrence 2.9 years later and underwent re‐resection, sparing the duodenum and inferior vena cava. (A, B) One year later, she again developed local recurrence in the right hemiabdomen with multifocal tumors, including a biopsy‐proven dedifferentiated component (tumor 1). After multidisciplinary team discussion, she underwent neoadjuvant systemic therapy, initially with a CDK inhibitor. She quickly progressed and was switched to single‐agent doxorubicin. She then underwent her fourth resection, which included *en bloc* right hemicolectomy, as well as removal of several peripancreatic tumors. (C) Post‐resection field with all gross disease removed. (D) Surgical resection specimens shown corresponding to pre‐operative imaging (tumors 1–3). CDK, cyclin‐dependent kinase; LPS, liposarcoma; WD, well‐differentiated. Figure courtesy of Steven Sun, MD and William W. Tseng, MD.

In a patient with recurrent disease, surgery is an important part of treatment, but the benefit versus risk and timing must be carefully considered in the context of other non‐surgical therapies as part of a multidisciplinary discussion (see case study depicted in Figure 2) [26]. In general, even for multiply recurrent disease, which is not uncommon in RP LPS, surgery should not be viewed as prohibitive or futile, as complete R0/R1 resection can still potentially provide the patient an extended disease‐free interval (DFI) [40]. Surgery in the context of recurrent disease is associated with specific additional technical considerations (e.g., adhesions or anatomic distortion due to prior surgery or treatment) [9]. In one study comparing surgery in recurrent versus primary RP sarcoma, overall morbidity (23% and 16.4%, respectively) and mortality (3% and 4.1%, respectively) were comparable [41]; however, the risks associated with surgery should be carefully considered for each individual patient by the treating center.

Importantly, for recurrent disease, disease biology becomes paramount in decision‐making [26]. Prognostic factors for surgery in recurrent disease include not only histologic subtype but also the number of organs resected in the primary surgery, age, number of recurrent tumors present, and the probability of incomplete resection [25]. Another important consideration is the DFI. Patients who have a short DFI after an adequate initial resection of primary disease or between recurrences typically derive less benefit with repeat surgery alone compared with those with a longer DFI [42]; therefore, multimodality treatment, with upfront non‐surgical therapy, may be indicated for patients with a short DFI (< 12 months) in some cases. The growth rate of the recurrent tumor may also be used to guide decision‐making and timing for surgery [9]. In one study of patients with RP LPS, those with a local recurrence growth rate > 0.9 cm per month did not benefit from surgery; thus, other non‐surgical (e.g., systemic) therapies should be considered for such patients [43]. However, there are scenarios where this principle may not apply [10]. In fact, there are situations in which surgery for disease recurrence is planned because of tumor growth and impending threat to critical organs or structures [9, 30]. The decision to proceed with surgery may also be impacted by tumor response to non‐surgical therapy.

### 4.4 Tumor Multifocality

Multifocality is defined as having two or more discontiguous tumors within the abdominal space, exclusive of visceral organ metastasis [44]. Multifocal disease is more common with recurrence but may also be encountered at primary presentation. For surgical planning, all sites of disease must be completely cleared to derive any benefit [44]. From a morbidity standpoint, planning must take into account potential additive post‐operative complications at multiple sites. It is currently unclear what the threshold number of tumors should be to preclude surgery [44]. However, in one study, clinical outcomes were significantly worse with seven or more tumors in both primary and recurrent disease [45].

## 5 Integration of Care

### 5.1 Multidisciplinary Team

The predominant and overwhelming clinical issue in RP LPS, both WD and DD, is that after surgery the risk of local recurrence is high [25]. This underscores the need for multimodality treatment to mitigate this issue; thus, having a multidisciplinary team is critical (See Endnote 1). This team should include a surgeon with specialized training in resection of RP sarcomas along with, at minimum, a medical oncologist, radiation oncologist, radiologist, and pathologist—all with expertise in the care of patients with sarcomas [12]. Continued dialog between the surgical oncologist and other specialists is critical for successful disease management [46]. The institution at which patients are treated is also important, as outcomes are significantly improved in patients with RP sarcomas who have undergone treatment at institutions with a high volume of cases compared with low‐volume institutions. One study suggested that the minimum threshold to maintain improved outcomes is 13 RP sarcoma cases per year, although it is important to note that many recognized referral centers have volumes of up to 24 cases per year [29, 47].

### 5.2 Radiation Therapy

RT is a treatment modality that can potentially improve local control in RP LPS. Apart from retrospective studies, including a multicenter study in RP LPS, the best available evidence to date comes from the STRASS (EORTC 62092) trial and STREXIT study [34, 35, 48]. The STRASS trial was a phase 3, multicenter, randomized study designed to evaluate whether pre‐operative RT combined with surgery improves outcomes compared with surgery alone in patients with primary, resectable retroperitoneal sarcoma (RPS) [35]. The primary endpoint was abdominal recurrence‐free survival (ARFS). After a median follow‐up of 43.1 months, the trial found no significant difference in ARFS between the two groups. While the overall results were negative, unplanned, and unpowered, exploratory analyses suggested that patients with WD LPS and low‐grade (G1–2) DD LPS might benefit from pre‐operative RT, showing improved ARFS in these subgroups. The STREXIT study was a retrospective observational analysis designed to complement the findings of the STRASS trial, which investigated the role of pre‐operative RT in patients with primary RPS [48]. While the STRASS trial did not demonstrate a significant benefit of pre‐operative RT in the overall RPS population, the STREXIT study aimed to explore potential benefits in specific subgroups by analyzing real‐world data. The STREXIT cohort included 831 adult patients treated with curative‐intent surgery for primary RPS between 2012 and 2017 at 10 centers participating in the STRASS trial [48]. In the pooled analysis of STRASS and matched STREXIT patients, pre‐operative RT was associated with improved ARFS in patients with LPS (hazard ratio [HR] 0.61; 95% confidence interval [CI], 0.42–0.89). Specifically, patients with WD LPS and G1–2 DD LPS showed a significant benefit from pre‐operative RT (HR 0.63; 95% CI, 0.40–0.97). No significant ARFS benefit was observed in patients with grade 3 DD LPS or leiomyosarcoma. It is important to note that there was no association between pre‐operative RT and overall survival or distant metastasis‐free survival in the overall cohort [48].

There were limitations of the STRASS study that merit consideration. A key issue was the lack of pre‐specified stratification by histologic subtype; analyses by subtype, such as those focusing on LPS, were conducted only post hoc [35]. Additionally, tumor grade data were missing for approximately a quarter of the enrolled patients. Among those assigned to preoperative RT, 25.6% did not adhere to the study protocol [49]. The trial also suffered from variability in surgical technique and differing definitions of unresectability across participating centers. Furthermore, patients who received RT experienced higher rates of intra‐operative complications and blood transfusion compared with those who underwent surgery alone [35].

Despite these limitations, taken together, the STRASS and STREXIT data suggest that pre‐operative RT may have a role in the management of patients with low‐grade LPS and, therefore, these patients merit discussion by a multidisciplinary tumor board. Novel strategies with neoadjuvant RT use the dose‐painting method, which limits treatment to an overall smaller area but, in turn, focuses the radiation dosing primarily to the “high‐risk” margins, which may be invaluable in treating RP LPS [50]. For select patients with RP LPS, RT in combination with surgery represents an opportunity for multimodality treatment.

### 5.3 Chemotherapy

Currently, an area of active investigation in RP LPS is systemic therapy, specifically in the neoadjuvant setting [51]. Systemic therapy given before surgery has several hypothetical advantages. Tumor size may be decreased to facilitate resection and potentially convert unresectable into resectable disease. Importantly, it could potentially eliminate both distant and local microscopic disease, leading to improved oncologic outcomes. Because of the frequently challenging nature of surgery in RP LPS and the potential for prolonged recovery, which may delay or preclude adjuvant therapy, the neoadjuvant approach is preferred.

To date, retrospective data in support of pre‐operative chemotherapy in RP LPS are inconsistent (See Endnote 1). Outcomes may even be worse in patients who received chemotherapy; however, this may be biased by patient selection, with patients in the neoadjuvant group having a worse disease status compared with those who received surgery up front [52]. The first prospective, phase 3 randomized trial for neoadjuvant systemic therapy in RP sarcoma, Surgery With or Without Neoadjuvant Chemotherapy in High‐Risk Retroperitoneal Sarcoma (STRASS2), is currently recruiting participants, with a primary objective to assess whether three cycles of pre‐operative chemotherapy followed by surgery can improve disease‐free survival compared with surgery alone [35, 53, 54]. The study includes patients with both RP leiomyosarcoma and DD LPS. For the latter, the chemotherapy regimen consists of three cycles of doxorubicin + ifosfamide. Anthracycline‐based regimens are established as the first‐line treatment for advanced or metastatic LPS [55, 56]. One potential limitation of the STRASS2 trial is its strict eligibility criteria, particularly for DD LPS given that needle biopsy may sometimes underestimate grade [53].

There are several other promising systemic therapy regimens being studied for advanced or metastatic LPS [7]. The application of these regimens—including targeted therapies, such as cyclin‐dependent kinase 4 and 6, MDM2 inhibitors, and immune checkpoint inhibitors—in the neoadjuvant setting represents a potential area of investigation in RP disease [7, 56, 57, 58, 59]. There are ongoing clinical trials on a number of neoadjuvant targeted therapies and immunotherapies specifically for the treatment of DD LPS. Additional work is being done to explore the potential of tumor‐infiltrating lymphocyte therapy for soft tissue sarcomas, including LPS [60].

## 6 Conclusion

While surgery is the standard treatment for RP LPS, the decision‐making process is complex, and there are clearly many opportunities for multimodality treatment. As the first step, recognition of the histologic subtype (WD versus DD) is critical, as this can potentially impact surgery and choice of multimodality treatment. Currently, non‐surgical therapies for RP LPS include RT and systemic therapy. Data from the STRASS trial are now available to help guide the implementation of RT for select patients in the neoadjuvant setting [35]. With the STRASS2 trial actively enrolling patients across the world [53], the benefit of neoadjuvant chemotherapy (specifically for DD) will soon be assessed.

Several areas within the RP LPS treatment landscape clearly merit continued investigation. Standardized criteria are needed for unresectability, based on both surgical and oncologic outcome data. These criteria must also account for LPS‐specific issues (e.g., WD versus DD, multifocality). Patient‐reported outcomes (PROs) for surgery and multimodality treatment, specifically in the context of shared decision‐making and patient expectations, are important to study, as such data provide more insight into the patient experience, particularly the overall impact of disease symptoms and treatment effects on patients' daily lives. During the STRASS trial, patient‐reported QoL assessment using the Quality of Life Questionnaire Core 30 (QLQ‐C30) was introduced via a protocol amendment; however, due to low compliance, the QLQ‐C30 data were not reported [35]. In another study, QoL was assessed in 127 patients with retroperitoneal soft tissue sarcoma who had undergone surgical resection using five validated questionnaires (EORTC QLQ‐C30, WEMWBS, FoP‐Q‐SF, PC‐PTSD, Pro‐CTCAE) [61]. Deficits in emotional and social functioning had a greater impact on QoL compared with physical limitations; however, QoL in this population was relatively unaffected, even in individuals who underwent multivisceral resection, experienced post‐operative complications, or had tumor recurrences. Insights gained through regular assessment of PROs can help identify areas where additional support for patients might be beneficial. The surgeries performed and outcomes (e.g., multiple recurrences) are arguably unique from other malignancies and even other sarcoma types. Studies must continue to identify actionable biomarkers that extend beyond histologic subtype to help guide treatment decision‐making. Finally, there is a critical need for novel systemic therapies (e.g., targeted therapies and immunotherapies) to be given alone or as an adjunct to surgery with RP LPS‐specific endpoints. Patient outcomes will only continue to improve with new insights gained from these areas of investigation.

## Author Contributions

**Steven Sun, Kenneth Cardona** and **William W. Tseng:** visualization; writing – original draft; review and editing.

## Conflicts of Interest

William W. Tseng reports membership on the Soft Tissue Sarcoma Panel for the National Comprehensive Cancer Network Inc. Steven Sun and Kenneth Cardona have nothing to disclose.

## Data Availability

Data sharing is not applicable to this article as no new data were created or analyzed.

## Endnotes

^1^
Referenced with permission from the NCCN Clinical Practice Guidelines in Oncology (NCCN Guidelines) for Soft Tissue Sarcoma V.4.2024. National Comprehensive Cancer Network, Inc. 2024. All rights reserved. Accessed November 25, 2024. To view the most recent and complete version of the guideline, go online to NCCN.org. NCCN makes no warranties of any kind whatsoever regarding their content, use or application and disclaims any responsibility for their application or use in any way.
